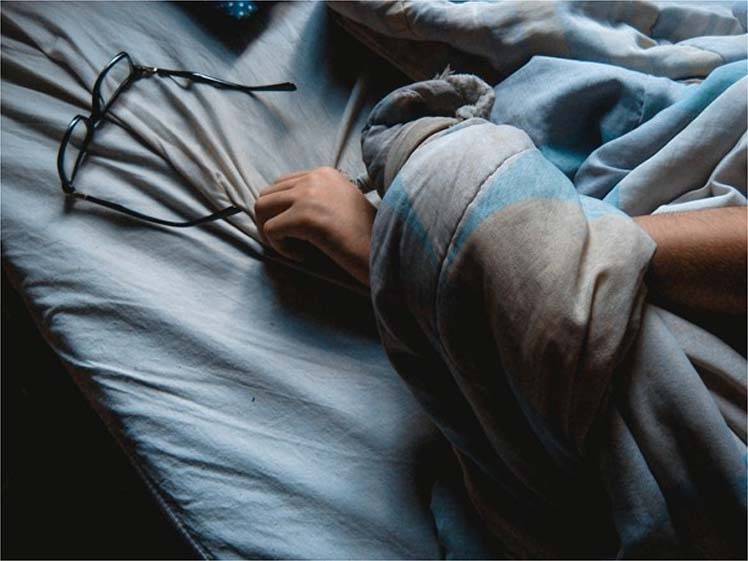# Sleep-wake cycles are disrupted by diseases that result in cytoplasmic crowding

**DOI:** 10.1038/s42003-020-01445-8

**Published:** 2020-11-19

**Authors:** Karli Montague-Cardoso

**Affiliations:** Communications Biology, https://www.nature.com/commsbio

## Abstract

It doesn’t take much to disrupt our sleep. Whilst we are aware of environmental factors that can disturb our circadian rhythms, the precise mechanisms that control molecular time cues have remained elusive. Beesley and co-workers demonstrate that diseases associated with cytoplasmic crowding affect the sleep-wake cycle. They also pinpoint a precise time-limiting step in the trafficking of the pacemaker protein PERIOD.

Circadian rhythms are physical, behavioural or mental processes that naturally follow a daily cycle. Perhaps the circadian rhythm we are most aware of is our sleep-wake cycle. Such cycles are driven by molecular time cues. The protein PERIOD (PER) is considered to be a major pacemaker protein due to its rhythm of accumulation and entry into the nucleus, which mediates the timing as well as the duration of feedback inhibition. We know from previous work that the manifestation of this feedback inhibition is a direct result of cytoplasmic trafficking of PER prior to its nuclear entry. What has remained elusive however, is the precise event that underlies how regulation of this pacemaker protein affects circadian timing.

In a recent study, Stephen Beesley and colleagues^1^ used a combination of in vitro, in vivo and mathematical modelling approaches to provide a clear understanding of how PER rhythms are generated, particularly in the context of disease. They identified a precise cellular event that regulates timing of the sleep-wake cycle. They demonstrated that sleep-wake cycles in mice were lengthened and considerably unstable when the trafficking of PER was disrupted by an increase in congestion in the cytoplasm in the form of autophagy-induced amino acid accumulation. They also provide compelling evidence that demonstrates that the timing and robustness of the circadian clock are precisely generated by delayed and collective phosphorylation of PER. They found that whilst active cytoskeletal transport of PER drives it to the perinucleus, once it has accumulated in there, it is likely that the time-limiting step is the subsequent dimerization and phosphorylation of PER that then enables entry into the nucleus. In other words, they successfully pinpointed a crucial nuclear gating step that dictates circadian rhythm timing.

This study provides an intriguing insight into how circadian rhythms are timed on a molecular level. In addition, it provides advances that help us to explain why sleep disorders may arise from different clinical conditions such as neurodegenerative and metabolic diseases, as well as aging, in which the cytoplasm becomes crowded.